# Huge giant cell tumor of the sacrum: A case report

**DOI:** 10.3892/ol.2014.1812

**Published:** 2014-01-17

**Authors:** LI-FENG QIN, DAN PENG, LI-HUA QIN, MIN XU, HAN FANG, QING ZHANG

**Affiliations:** 1 Department of Orthopedics, The Second Xiang-Ya Hospital, Central South University, Changsha, Hunan 410010, P.R. China; 2 Department of Gynecology, Nursing School of Hunan University of Chinese Medicine, Changsha, Hunan 410208, P.R. China; 3 Intensive Care Unit, The Second Xiang-Ya Hospital, Central South University, Changsha, Hunan 410008, P.R. China; 4 Department of Cardiovascular Disease, Xiang-Ya Hospital Central South University, Changsha, Hunan 410008, P.R. China

**Keywords:** giant cell tumor, chordoma, S2 nerve root, sacral tumor, rectum, urinary and bowel function

## Abstract

The current report describes the case of a 29-year-old female with a sacral giant cell tumor (GCT) during pregnancy. Originally, the patient presented with severe pain in the lumbosacral region, radiating posterolaterally from the lumbar spine into the bilateral thigh and subsequently, into the bilateral crus posterolaterally. Plain X-rays, computed tomography and magnetic resonance imaging showed osteolytic destruction of the sacrococcygeal bones and a huge soft-tissue mass with features of a chordoma. The patient underwent a partial en bloc sacrectomy (partial S1 and completely below) and curettage for tumors located at the sacroiliac joint and underlying left ilium, with bilateral internal iliac arteries ligated to control intraoperative hemorrhage. The patient’s bilateral S2 nerve roots were killed. The diagnosis of conventional GCT was determined based on the histopathological examination of the resected specimen. Urinary and bowel functions were recovered by exercising.

## Introduction

Giant cell tumors (GCTs) of the bone are expansile osteolytic tumors in young adults that usually occur at the end of the long bones. Although only 6% of GCTs occur in the sacrum, GCTs are the second most common type of primary tumor involving bone in the sacrum (1,2). Recently, certain authors have claimed that parathyroid hormone-related protein may act locally within GCTs and be significant in the pathogenesis of these tumors (3). It is rare to identify a GCT complicating pregnancy, and a correlation between tumor growth and pregnancy has not yet been clarified.

Sacral GCTs tend to be clinically silent during their initial stages of development and cause few symptoms until they achieve an extremely large size, particularly when occurring during pregnancy. A number of patients have been initially misdiagnosed with prolapses of lumbar intervertebral discs, chordomas or other tumors. Lumbosacral magnetic resonance imaging (MRI) is useful for the diagnosis of these conditions. Although histologically benign, these osteolytic expansive tumors are locally aggressive, and the local recurrence rate in the sacrum is higher than recurrence rates at other skeletal locations. In addition, sacral GCTs have been shown to metastasize (4). Treatment options include radiation therapy, surgery (such as intralesional curettage and wide excision by complete or partial en bloc sacrectomy), surgery plus adjuvant treatment and serial arterial embolization (5,6). More recently, the bisphosphonate zoledronic acid (7) and interferon α-2b (8) have been reported to be effective and safe adjuvant treatments in patients with spinal GCT, particularly in those with recurrent and metastatic tumors.

## Case report

A 29-year-old female experienced severe pain in the lumbosacral region on the seventh day after the delivery of a child. The pain was initially considered to be due to pregnancy and delivery and no further examination was performed. However, the patient continued to experience persistent pain and a burning sensation in the lumbosacral region, particularly at night. The pain radiated from the lumbar spine into each thigh posterolaterally and subsequently, into the bilateral crus posterolaterally. The patient also experienced a change of bowel habits. Eight months later, the patient presented at the XinNing Country Sunshine Hospital (Shaoyang, China) with continuing discomfort and pain in the lumbosacral region. MRI revealed a huge tumor mass (95×70×90 mm in size) involving the sacrococcygeal region, indicating a chordoma.

One week later, the patient was transferred to the Hunan Xiang-ya Second Hospital (Changsha, China). The patient had not menstruated during the eight months since delivery. A physical examination upon admission showed that the patient was positive for Lasegue’s sign at 70 degrees on each side. The patient experienced pain on percussion of the sacrum and the temperature of the sacral skin was high. Rectal examination revealed a huge presacral and toughening mass of 9×7 cm in size. The mass exhibited a regular surface, was firm in consistency, was not tender and was fixed to the sacrum. Blood biochemistry analysis revealed that the alkaline phosphatase, serum iron and C-reactive protein concentrations and the erythrocyte sedimentation rate were all within normal ranges. Chest X-ray observations were normal.

**Figure 1:**
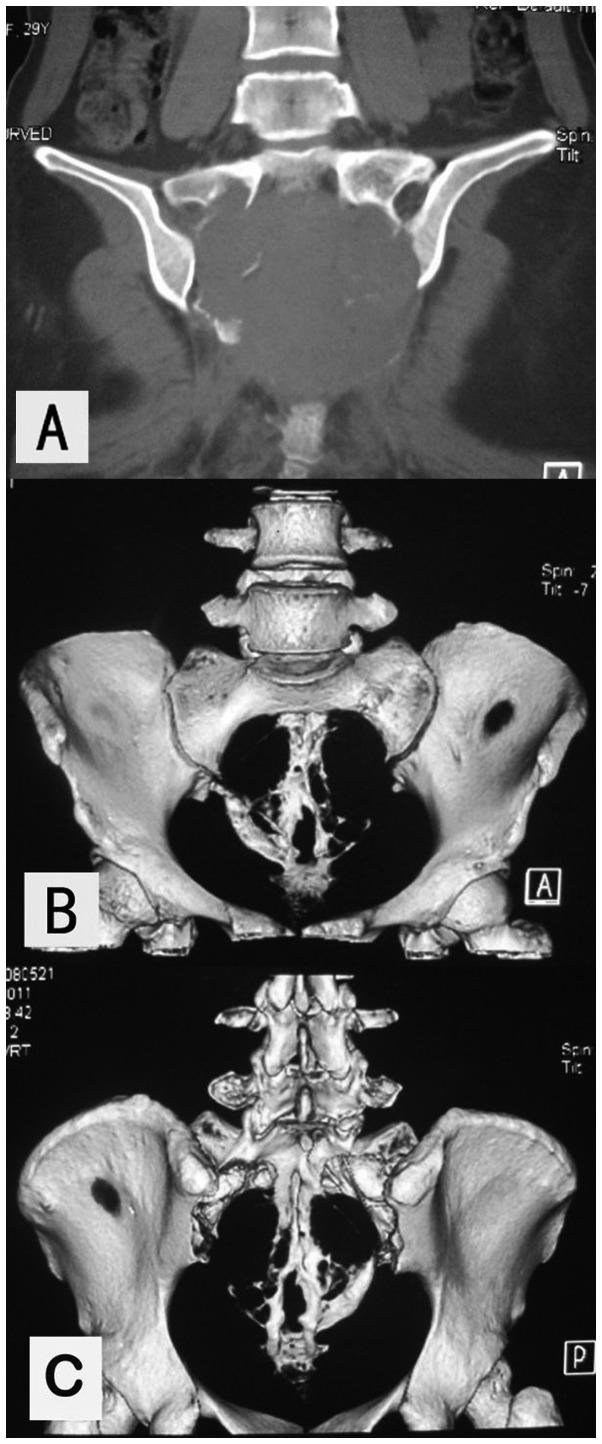
(A) Computed tomography (CT) scan and (B and C) three-dimensional reconstruction showing osteolytic destruction of the sacrum and a large soft-tissue mass involving the underlying left ilium. (B) Three-dimensional scan viewed from the front. (C) Three-dimensional scan viewed from behind. The sacral canal was enlarged and the left medial ilium (near sacroiliac joints) was destroyed.

**Figure 2:**
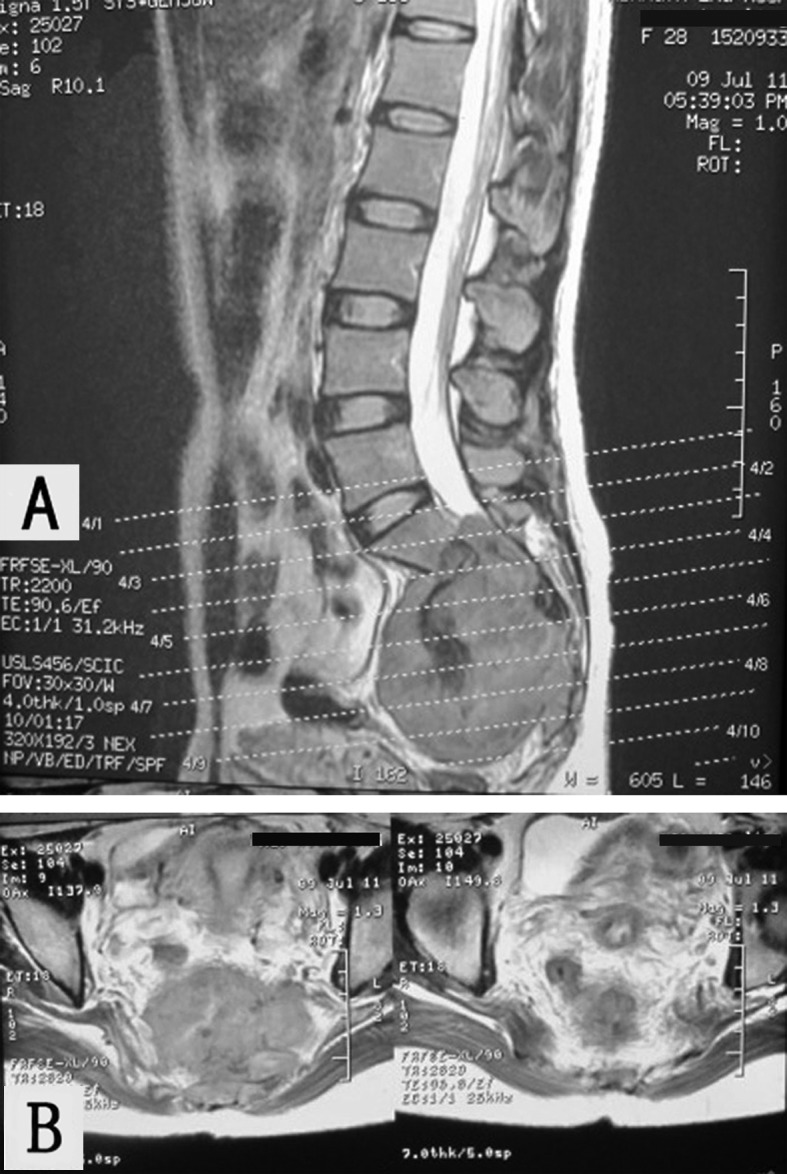
(A and B) Magnetic resonance images (MRI) showing the destruction of the sacrococcygeal bones and a huge soft-tissue mass. On T1- and T2-weighted images, the lesion showed large patches of mixed signal shadows, which followed the plane of the first sacral vertebra. (A) The boundary between the mass and the rectum was unclear. (B) Upon enhanced scanning, the lesions appeared more pronounced.

Plain X-rays of the lumbar spine, sacrum and pelvis revealed a large amount of expansile osteolytic destruction of the sacrum involving the upper foramina and right iliac bone, as well as a pathological fracture. The lytic lesions were surrounded by a soft-tissue mass, without peripheral bone sclerosis. Three-dimensional reconstructions of computed tomography (CT) scans (Fig. 1) and MRI (Fig. 2) revealed osteolytic destruction of the sacrococcygeal bones, including parts of the first sacral vertebra and underlying left internal ilium, as well as a huge soft-tissue mass measuring 95×75 mm in size. The sacral canal was enlarged. The lesion showed large patches of mixed signal shadows on T1- and T2-weighted images, which followed the plane of the first sacral vertebra. An unclear boundary was identified between the mass and rectum. Upon enhanced scanning, the lesions appeared more pronounced. The results of plain X-ray, CT and MRI indicated a diagnosis of a chordoma.

The patient was treated surgically. The two sciatic nerves were contained within the tumor. In addition, the tumor had invaded the left sacroiliac joint and partially invaded the left iliac surface. The thecal sac had been cut inferiorly to the S1 vertebrae. Therefore, the thecal sac was ligated at the S1–S2 level, the sacrum was cut with osteotomies under the S1 superior border, keeping a thin layer of bone and ilium linked laterally, and the sciatic nerves were separated from the tumor. The tumor was resected en bloc as a section greater than 11×11×7 cm in size, along with the sacral nerves, and the pelvic ring was reconstructed using an arc pelvic reconstruction plate connected to the bilateral ilia. The remaining sectional S1 vertebrae received two massive allografts, which were fixed with screws. The patient required transfusions with 1,950 ml of enriched red blood cell suspension and 900 ml of platelets during surgery. The patient was diagnosed with a conventional GCT based on the histopathological examination of the resected specimen.

**Figure 3:**
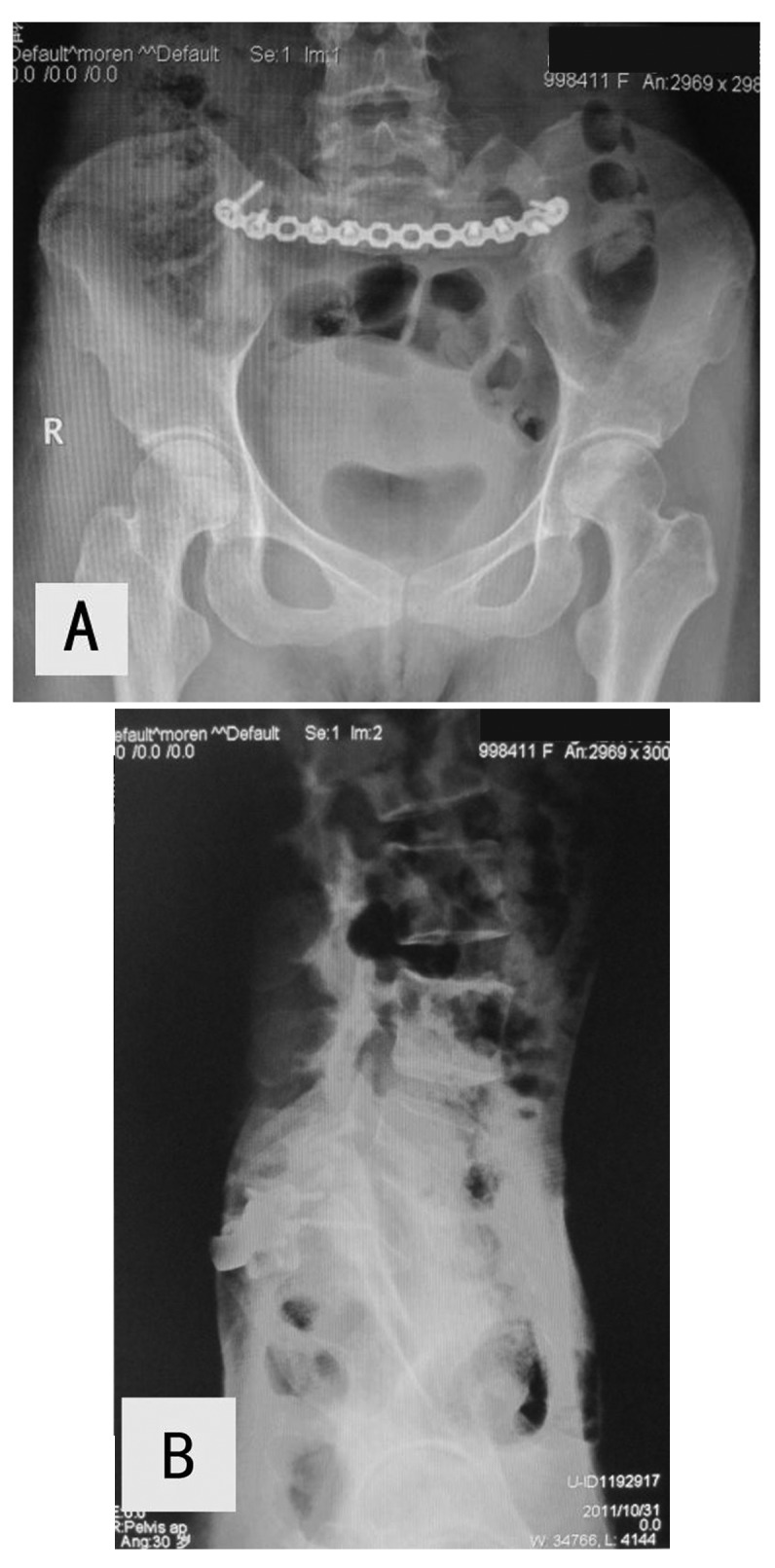
Plain X-ray of the (A) pelvic and (B) sacroiliac regions six months after surgery, neither of which exhibited any looseness or fractures of internal fixation or new bone destruction.

The patient and the patient’s family were informed that a colostomy was likely to be required if the functions of the bladder and anal sphincter were not recovered. Therefore, the patient performed exercises to strengthen the function of the bladder, anal sphincter and muscles of the lower limbs in bed 600 times per day. The patient was able to pass urine two months postoperatively and stool three months postoperatively. Plain X-rays of the pelvic and sacroiliac regions at three months showed no looseness or fractures of internal fixation and no new bone destruction (Fig. 3). Six months after surgery, the patient was able to walk with a cane and had control of the urinary bladder and anal sphincter. One year later, the patient was able to walk without a cane and had good control of the urinary bladder and anal sphincter. Although the two bilateral S2 nerve roots were lost, the patient recovered urinary and bowel function by exercising. The patient provided written informed consent.

## Discussion

The detection of a GCT may be delayed, since tumor-related pain is frequently misinterpreted as a symptom of pregnancy, as observed in the present patient. Care must be taken not to overlook the possibility of a tumor in the sacrum or innominate bone during pregnancy.

Although surgery remains the first-line treatment for sacral tumors, their difficult location and huge size, as well as the possibility of life-threatening intraoperative bleeding, make surgery difficult (9). In addition, microscopic lesions may remain when the sacral nerve roots are preserved, whereas bowel and bladder function may be compromised when the sacral nerve roots are not preserved. Other problems include difficulties reconstructing the stability of the pelvis and spine and local recurrence. Notably, local recurrence rates have been found to approach 50% in patients who do not undergo surgery with wide margins, by complete or partial en bloc sacrectomy (10). Although surgery with wide margins results in a significant decrease in the local recurrence rate, wide resection often requires the sacral nerve roots to be sacrificed. In addition, effective control of massive bleeding and a reconstruction of pelvic and spinal stability is required, particularly since a number of GCTs involve the upper sacral segments, frequently crossing the midline and even the sacroiliac joint (4,6). The surgical technique used in the current study consisted of curettage for tumors located at the sacroiliac joint and underlying left ilium and a partial en bloc sacrectomy (partial S1 and completely below). The pelvic ring was reconstructed using an arc pelvic reconstruction plate and two massive allografts.

Arterial embolization (5), followed by complete occlusion of the artery, may minimize intraoperative bleeding and aid in directly resecting the tumor and decreasing the local recurrence rate (11). Therefore, in the present study, the two internal iliac arteries were ligated to control intraoperative hemorrhage. If the two S2 nerve roots may be preserved, approximately half of patients are likely to retain bowel and bladder function. However, bowel and bladder function are lost if only the unilateral S2 nerve root is spared (6). Unilateral resection of the sacral roots or preservation of at least one S3 nerve root upon bilateral resection has been found to preserve bowel and bladder function in the majority of patients (12). Although the patient of the current study lost the two S2 nerve roots, their desire to recover bowel and bladder function was extremely strong. Therefore, the patient performed exercises to strengthen the function of the bladder and anal sphincter and increase muscle strength in the lower limbs.

Local malignant transformation has been reported to occur in ≤16% of patients with primary GCTs, with 1–9% of patients showing lung metastases of GCTs of the bone (6,13). To date, the present patient has shown no evidence of local recurrence on pelvic X-rays and no lung metastases on chest X-rays.

In conclusion, complaints, such as pain, discomfort or numbness around the pelvis, particularly during pregnancy, may be the direct result of a tumor in the pelvic bone. The current study described a patient who, despite the loss of the bilateral S2 nerve roots, recovered function of the urinary bladder and anal sphincter by exercising.
